# 2-(2,6-Dichloro­benz­yl)pyrrolidine-1,3-dione

**DOI:** 10.1107/S1600536809024970

**Published:** 2009-07-04

**Authors:** Peng-Mian Huang, Xiao-Chun Wang

**Affiliations:** a College of Chemistry & Bioengineering, Changsha University of Science & Technology, Changsha 410076, People’s Republic of China; b Department of Clinical Laboratory, XiangYa Medical College of Central, South University, Changsha 410013, People’s Republic of China

## Abstract

In the title compound, C_11_H_9_Cl_2_NO_2_, the dihedral anngle between the mean planes of the aromatic ring and the twisted pyrrolidinedione ring is 79.98 (9)°.

## Related literature

For the synthesis, see: Duan *et al.* (2005 ▶). For the pharmaceutical properties of pyrrolidine-2,5-dione derivatives, see: Obniska *et al.* (2009 ▶).
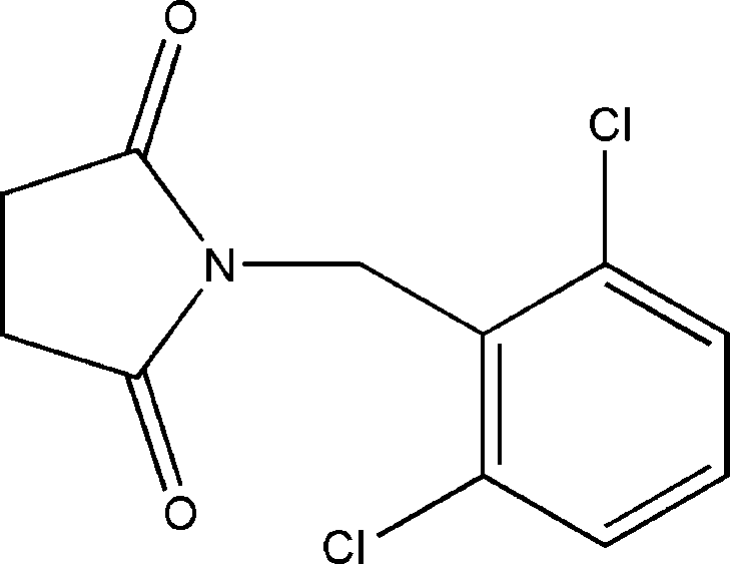


## Experimental

### 

#### Crystal data

C_11_H_9_Cl_2_NO_2_
*M* *_r_* = 258.09
Orthorhombic, 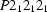

*a* = 4.8057 (5) Å
*b* = 9.4388 (8) Å
*c* = 23.936 (2) Å
*V* = 1085.74 (18) Å^3^
*Z* = 4
Mo *K*α radiation
μ = 0.58 mm^−1^
*T* = 113 K
0.14 × 0.12 × 0.10 mm

#### Data collection

Rigaku Saturn CCD area-detector diffractometer
Absorption correction: multi-scan (*CrystalClear*; Rigaku/MSC, 2005 ▶) *T*_min_ = 0.923, *T*_max_ = 0.944
7528 measured reflections
2562 independent reflections
2400 reflections with *I* > 2σ(*I*)
*R*_int_ = 0.029

#### Refinement

*R*[*F* ^2^ > 2σ(*F* ^2^)] = 0.031
*wR*(*F* ^2^) = 0.073
*S* = 1.09
2562 reflections
146 parameters
H-atom parameters constrained
Δρ_max_ = 0.28 e Å^−3^
Δρ_min_ = −0.27 e Å^−3^
Absolute structure: Flack (1983 ▶), 1017 Friedel pairs
Flack parameter: 0.01 (6)

### 

Data collection: *CrystalClear* (Rigaku/MSC, 2005 ▶); cell refinement: *CrystalClear*; data reduction: *CrystalClear*; program(s) used to solve structure: *SHELXS97* (Sheldrick, 2008 ▶); program(s) used to refine structure: *SHELXL97* (Sheldrick, 2008 ▶); molecular graphics: *SHELXTL* (Sheldrick, 2008 ▶); software used to prepare material for publication: *CrystalStructure* (Rigaku/MSC, 2005 ▶).

## Supplementary Material

Crystal structure: contains datablocks I, global. DOI: 10.1107/S1600536809024970/hb5015sup1.cif

Structure factors: contains datablocks I. DOI: 10.1107/S1600536809024970/hb5015Isup2.hkl

Additional supplementary materials:  crystallographic information; 3D view; checkCIF report

## Figures and Tables

**Fig. 1.**
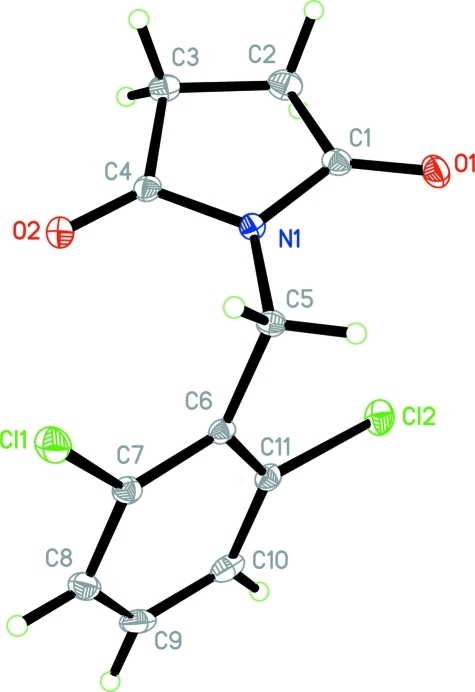
Molecular structure of the title compound with displacement ellipsoids drawn at the 30% probability level. H atoms are presented as spheres of arbitrary radius.

## supplementary crystallographic information

### Comment

The derivatives of pyrrolidine-2,5-dione possess valuable pharmaceutical properties (Obniska *et al.*, 2009). In this paper, synthesis and the crystal structure of the title compound, (I), Fig 1, is reported.

### Experimental

The title compound was prepared according to the procedure of Duan *et al.* (2005).  The title compound (40 mg) was dissolved in a mixture of chloroform (5 ml) and ethanol (3 ml) and the solution was kept at room temperature for 15 days. Evaporation of the solution gave colourless blocks of (I).

### Refinement

All H atoms were included in the idealized positions (C—H = 0.93–0.99Å) and refined as riding with and refined in a riding with *U*_iso_(H) = 1.2*U*_eq_(C).

### Crystal data

**Table:**

C_11_H_9_Cl_2_NO_2_	*D*_x_ = 1.579 Mg m^−^^3^
*M**_r_* = 258.09	Melting point = 409–411 K
Orthorhombic, *P*2_1_2_1_2_1_	Mo *K*α radiation, λ = 0.71070 Å
Hall symbol: P 2ac 2ab	Cell parameters from 2881 reflections
*a* = 4.8057 (5) Å	θ = 1.7–27.9°
*b* = 9.4388 (8) Å	µ = 0.58 mm^−^^1^
*c* = 23.936 (2) Å	*T* = 113 K
*V* = 1085.74 (18) Å^3^	Block, colorless
*Z* = 4	0.14 × 0.12 × 0.10 mm
*F*(000) = 528	

### Data collection

**Table:**

Rigaku Saturn CCD area-detector diffractometer	2562 independent reflections
Radiation source: rotating anode	2400 reflections with *I* > 2σ(*I*)
confocal	*R*_int_ = 0.029
Detector resolution: 7.31 pixels mm^-1^	θ_max_ = 27.9°, θ_min_ = 1.7°
ω and φ scans	*h* = −4→6
Absorption correction: multi-scan (*CrystalClear*; Rigaku/MSC, 2005)	*k* = −12→12
*T*_min_ = 0.923, *T*_max_ = 0.944	*l* = −30→31
7528 measured reflections	

### Refinement

**Table:**

Refinement on *F*^2^	Hydrogen site location: inferred from neighbouring sites
Least-squares matrix: full	H-atom parameters constrained
*R*[*F*^2^ > 2σ(*F*^2^)] = 0.031	*w* = 1/[σ^2^(*F*_o_^2^) + (0.0337*P*)^2^ + 0.1572*P*] where *P* = (*F*_o_^2^ + 2*F*_c_^2^)/3
*wR*(*F*^2^) = 0.073	(Δ/σ)_max_ < 0.001
*S* = 1.09	Δρ_max_ = 0.28 e Å^−^^3^
2562 reflections	Δρ_min_ = −0.27 e Å^−^^3^
146 parameters	Extinction correction: *SHELXL97* (Sheldrick, 2008), Fc^*^=kFc[1+0.001xFc^2^λ^3^/sin(2θ)]^-1/4^
0 restraints	Extinction coefficient: 0.027 (3)
Primary atom site location: structure-invariant direct methods	Absolute structure: Flack (1983), 1017 Friedel pairs
Secondary atom site location: difference Fourier map	Flack parameter: 0.01 (6)

### Special details

**Table:**

Geometry. All esds (except the esd in the dihedral angle between two l.s. planes) are estimated using the full covariance matrix. The cell esds are taken into account individually in the estimation of esds in distances, angles and torsion angles; correlations between esds in cell parameters are only used when they are defined by crystal symmetry. An approximate (isotropic) treatment of cell esds is used for estimating esds involving l.s. planes.
Refinement. Refinement of F^2^ against ALL reflections. The weighted R-factor wR and goodness of fit S are based on F^2^, conventional R-factors R are based on F, with F set to zero for negative F^2^. The threshold expression of F^2^ > 2sigma(F^2^) is used only for calculating R-factors(gt) etc. and is not relevant to the choice of reflections for refinement. R-factors based on F^2^ are statistically about twice as large as those based on F, and R- factors based on ALL data will be even larger.

### Fractional atomic coordinates and isotropic or equivalent isotropic displacement parameters (Å^2^)

**Table:**

	*x*	*y*	*z*	*U*_iso_*/*U*_eq_	
Cl1	0.00935 (11)	−0.15827 (4)	0.039298 (15)	0.02779 (12)	
Cl2	−0.01306 (10)	0.14810 (5)	0.231345 (15)	0.02661 (12)	
O1	−0.4371 (3)	0.35252 (13)	0.15425 (5)	0.0256 (3)	
O2	0.2345 (3)	0.17916 (13)	0.03882 (5)	0.0258 (3)	
N1	−0.1077 (3)	0.23490 (14)	0.10180 (5)	0.0172 (3)	
C1	−0.2311 (4)	0.35434 (18)	0.12493 (6)	0.0201 (3)	
C2	−0.0631 (4)	0.48127 (18)	0.10689 (8)	0.0276 (4)	
H2A	0.0514	0.5174	0.1382	0.033*	
H2B	−0.1863	0.5582	0.0936	0.033*	
C3	0.1211 (4)	0.42692 (18)	0.05958 (7)	0.0222 (4)	
H3A	0.0520	0.4604	0.0229	0.027*	
H3B	0.3156	0.4591	0.0645	0.027*	
C4	0.1017 (4)	0.26745 (17)	0.06376 (6)	0.0186 (3)	
C5	−0.2043 (4)	0.09082 (17)	0.11224 (6)	0.0177 (3)	
H5A	−0.2735	0.0496	0.0768	0.021*	
H5B	−0.3619	0.0939	0.1389	0.021*	
C6	0.0205 (4)	−0.00365 (15)	0.13563 (6)	0.0160 (3)	
C7	0.1336 (4)	−0.11631 (17)	0.10562 (7)	0.0193 (3)	
C8	0.3417 (4)	−0.20312 (18)	0.12690 (8)	0.0255 (4)	
H8	0.4149	−0.2783	0.1050	0.031*	
C9	0.4416 (4)	−0.17880 (19)	0.18054 (8)	0.0283 (4)	
H9	0.5851	−0.2370	0.1953	0.034*	
C10	0.3327 (4)	−0.0703 (2)	0.21228 (7)	0.0253 (4)	
H10	0.3987	−0.0538	0.2491	0.030*	
C11	0.1251 (4)	0.01445 (17)	0.18967 (7)	0.0195 (3)	

### Atomic displacement parameters (Å^2^)

**Table:**

	*U*^11^	*U*^22^	*U*^33^	*U*^12^	*U*^13^	*U*^23^
Cl1	0.0382 (3)	0.0230 (2)	0.02216 (19)	0.0038 (2)	−0.0022 (2)	−0.00636 (14)
Cl2	0.0287 (2)	0.0340 (2)	0.01716 (18)	0.0004 (2)	−0.00099 (17)	−0.00597 (15)
O1	0.0261 (7)	0.0286 (6)	0.0223 (5)	0.0089 (6)	0.0024 (5)	−0.0028 (5)
O2	0.0255 (7)	0.0264 (7)	0.0254 (6)	0.0043 (5)	0.0073 (5)	0.0028 (5)
N1	0.0175 (7)	0.0171 (6)	0.0171 (6)	0.0008 (6)	0.0003 (6)	−0.0001 (5)
C1	0.0224 (9)	0.0194 (8)	0.0186 (7)	0.0029 (7)	−0.0033 (6)	−0.0012 (6)
C2	0.0315 (11)	0.0185 (7)	0.0329 (9)	−0.0017 (8)	−0.0009 (8)	−0.0001 (7)
C3	0.0218 (9)	0.0207 (8)	0.0242 (8)	−0.0039 (7)	−0.0037 (7)	0.0034 (7)
C4	0.0177 (8)	0.0227 (8)	0.0155 (7)	0.0009 (7)	−0.0020 (6)	0.0018 (6)
C5	0.0188 (8)	0.0153 (7)	0.0189 (7)	−0.0005 (6)	−0.0016 (7)	−0.0002 (6)
C6	0.0141 (8)	0.0167 (7)	0.0174 (6)	−0.0022 (7)	−0.0003 (6)	0.0030 (5)
C7	0.0185 (8)	0.0183 (7)	0.0210 (7)	−0.0012 (7)	−0.0001 (7)	0.0023 (6)
C8	0.0231 (10)	0.0179 (8)	0.0354 (9)	0.0017 (7)	0.0043 (8)	0.0042 (7)
C9	0.0215 (10)	0.0245 (9)	0.0388 (10)	0.0014 (8)	−0.0007 (8)	0.0156 (7)
C10	0.0221 (10)	0.0303 (9)	0.0235 (8)	−0.0061 (8)	−0.0035 (7)	0.0109 (7)
C11	0.0172 (8)	0.0223 (8)	0.0190 (7)	−0.0021 (7)	0.0014 (7)	0.0031 (6)

### Geometric parameters (Å, °)

**Table:**

Cl1—C7	1.7417 (17)	C3—H3B	0.9900
Cl2—C11	1.7400 (17)	C5—C6	1.509 (2)
O1—C1	1.213 (2)	C5—H5A	0.9900
O2—C4	1.208 (2)	C5—H5B	0.9900
N1—C1	1.389 (2)	C6—C7	1.394 (2)
N1—C4	1.391 (2)	C6—C11	1.398 (2)
N1—C5	1.459 (2)	C7—C8	1.390 (2)
C1—C2	1.508 (2)	C8—C9	1.390 (3)
C2—C3	1.526 (3)	C8—H8	0.9500
C2—H2A	0.9900	C9—C10	1.378 (3)
C2—H2B	0.9900	C9—H9	0.9500
C3—C4	1.511 (2)	C10—C11	1.389 (2)
C3—H3A	0.9900	C10—H10	0.9500
			
C1—N1—C4	112.99 (14)	C6—C5—H5A	109.0
C1—N1—C5	123.52 (14)	N1—C5—H5B	109.0
C4—N1—C5	123.25 (13)	C6—C5—H5B	109.0
O1—C1—N1	124.58 (16)	H5A—C5—H5B	107.8
O1—C1—C2	127.86 (16)	C7—C6—C11	115.46 (15)
N1—C1—C2	107.56 (14)	C7—C6—C5	122.61 (14)
C1—C2—C3	104.84 (14)	C11—C6—C5	121.91 (14)
C1—C2—H2A	110.8	C8—C7—C6	122.79 (16)
C3—C2—H2A	110.8	C8—C7—Cl1	116.53 (14)
C1—C2—H2B	110.8	C6—C7—Cl1	120.64 (13)
C3—C2—H2B	110.8	C7—C8—C9	119.33 (17)
H2A—C2—H2B	108.9	C7—C8—H8	120.3
C4—C3—C2	104.48 (14)	C9—C8—H8	120.3
C4—C3—H3A	110.9	C10—C9—C8	120.04 (16)
C2—C3—H3A	110.9	C10—C9—H9	120.0
C4—C3—H3B	110.9	C8—C9—H9	120.0
C2—C3—H3B	110.9	C9—C10—C11	119.09 (16)
H3A—C3—H3B	108.9	C9—C10—H10	120.5
O2—C4—N1	123.60 (15)	C11—C10—H10	120.5
O2—C4—C3	128.46 (16)	C10—C11—C6	123.25 (16)
N1—C4—C3	107.93 (14)	C10—C11—Cl2	117.94 (13)
N1—C5—C6	112.74 (14)	C6—C11—Cl2	118.80 (13)
N1—C5—H5A	109.0		
			
C4—N1—C1—O1	171.59 (15)	N1—C5—C6—C11	69.50 (19)
C5—N1—C1—O1	−3.0 (2)	C11—C6—C7—C8	−1.7 (2)
C4—N1—C1—C2	−8.48 (18)	C5—C6—C7—C8	179.66 (15)
C5—N1—C1—C2	176.92 (14)	C11—C6—C7—Cl1	176.23 (12)
O1—C1—C2—C3	−165.92 (16)	C5—C6—C7—Cl1	−2.4 (2)
N1—C1—C2—C3	14.14 (18)	C6—C7—C8—C9	0.7 (3)
C1—C2—C3—C4	−14.26 (18)	Cl1—C7—C8—C9	−177.37 (14)
C1—N1—C4—O2	−179.73 (16)	C7—C8—C9—C10	0.6 (3)
C5—N1—C4—O2	−5.1 (3)	C8—C9—C10—C11	−0.7 (3)
C1—N1—C4—C3	−1.03 (18)	C9—C10—C11—C6	−0.5 (3)
C5—N1—C4—C3	173.59 (14)	C9—C10—C11—Cl2	178.68 (13)
C2—C3—C4—O2	−171.54 (18)	C7—C6—C11—C10	1.7 (2)
C2—C3—C4—N1	9.84 (18)	C5—C6—C11—C10	−179.73 (16)
C1—N1—C5—C6	−123.48 (16)	C7—C6—C11—Cl2	−177.52 (13)
C4—N1—C5—C6	62.46 (19)	C5—C6—C11—Cl2	1.1 (2)
N1—C5—C6—C7	−111.97 (17)		
